# Supplementary material for: Multi-focus averaging for multiple scattering suppression in optical coherence tomography
Source: Biomed Opt Express. 2023 Aug 24;14(9):4828–44. doi: 10.1364/BOE.493706 (PMC10545188; doi:10.1364/BOE.493706)
Supplement: Supplementary file 1 [file boe-14-9-4828-s001.pdf]

Supplemental Document

Biomedical Optics EXPRESS

Multi-focus averaging for multiple scattering suppression in optical coherence tomography: supplement

LIDA ZHU,[1] SHUICHI MAKITA,[1] JUNYA TAMAOKI,[2] ANTONIA LICHTENEGGER,[1,3] YIHENG LIM,[1] YIQIANG ZHU,[1] MAKOTO KOBAYASHI,[2] AND YOSHIAKI YASUNO[1,*]

[1]*Computational Optics Group, University of Tsukuba, Tsukuba, Ibaraki, Japan*
[2]*Department of Molecular and Developmental Biology, Institute of Medicine, University of Tsukuba, Japan*
[3]*Center for Medical Physics and Biomedical Engineering, Medical University of Vienna, Vienna, Austria*
https://optics.bk.tsukuba.ac.jp/COG/
[*]*yoshiaki.yasuno@cog-labs.org*

This supplement published with Optica Publishing Group on 24 August 2023 by The Authors under the terms of the Creative Commons Attribution 4.0 License in the format provided by the authors and unedited. Further distribution of this work must maintain attribution to the author(s) and the published article's title, journal citation, and DOI.

Supplement DOI: https://doi.org/10.6084/m9.figshare.23851674

Parent Article DOI: https://doi.org/10.1364/BOE.493706

Multi-focus averaging for multiple scattering suppression in optical coherence tomography: supplemental document

This file supplements Fig. 3 by showing additional intensity depth profiles using the A-line sets from three different locations. All of the results draw the same conclusion that the MFA profiles show more intensity reduction than the SFA and single acquisition profiles in the deep regions.

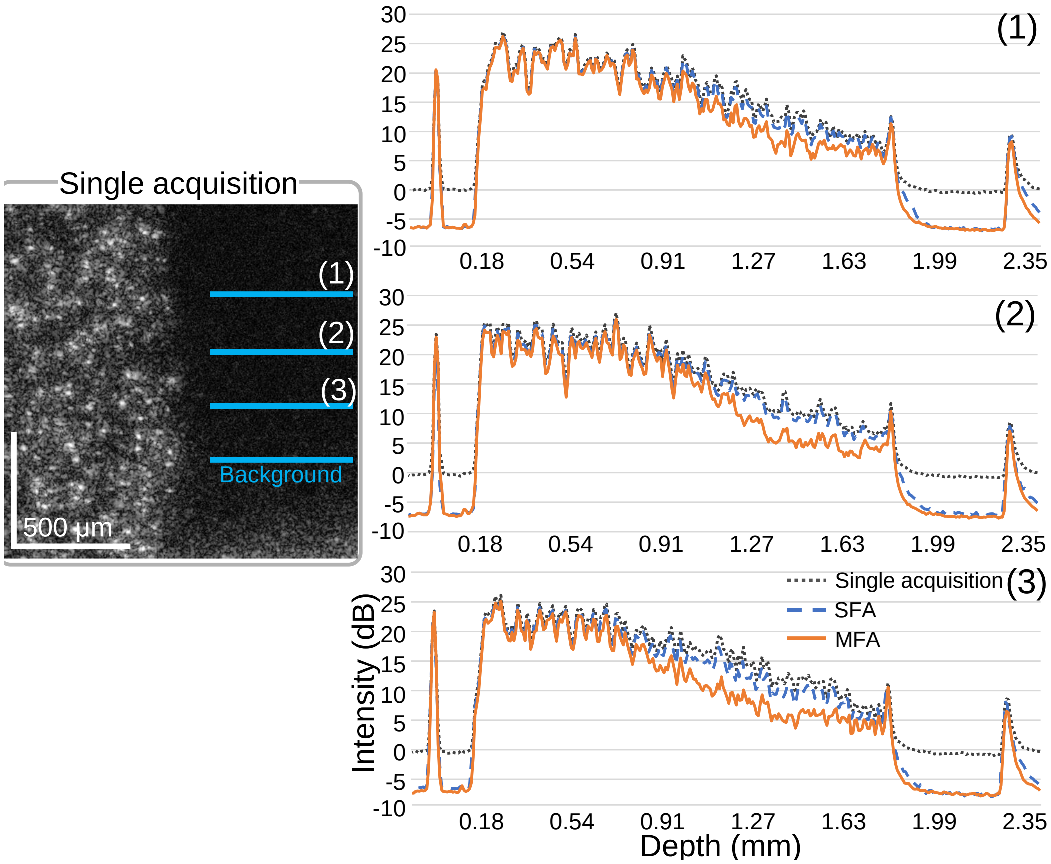

Fig. S1. Intensity depth profiles obtained using 2 × 200 A-lines sets at three different locations as indicated by the blue lines. The phantom data is identical to the data used in Fig. 3, and the *en-face* image is identical to Fig. 3(d). From these profiles computed at different locations, we can conclude that MFA reduces the signal intensity in the deep regions, which could be benefited from MS reduction.
